# Sequence conservation of mitochondrial (mt)DNA during expansion of clonal mammary epithelial populations suggests a common mtDNA template in CzechII mice

**DOI:** 10.18632/oncotarget.27429

**Published:** 2020-01-14

**Authors:** Jabril R. Johnson, Justin B. Lack, Corinne A. Boulanger, Lauren E. Ragle, Gilbert H. Smith

**Affiliations:** ^1^Mammary Stem Cell Biology Section, National Cancer Institute, Bethesda, MD 20892, USA; ^2^Bioinformatics Manager/Lead, NIAID Collaborative Bioinformatics Resource (NCBR) Frederick National Laboratory for Cancer Research, Leidos Biomedical Research, Inc., Bethesda, MD 20894, USA; ^3^Department of Population Sciences, City of Hope, Duarte, CA 91107, USA

**Keywords:** mitochondrial DNA, next-generation sequencing, clonal expansion, mammary cancer

## Abstract

One major foundation of cancer etiology is the process of clonal expansion. The mechanisms underlying the complex process of a single cell leading to a clonal dominant tumor, are poorly understood. Our study aims to analyze mitochondrial DNA (mtDNA) for somatic single nucleotide polymorphisms (SNPs) variants, to determine if they are conserved throughout clonal expansion in mammary tissues and tumors. To test this hypothesis, we took advantage of a mouse mammary tumor virus (MMTV)-infected mouse model (CzechII). CzechII mouse mtDNA was extracted, from snap-frozen normal, hyperplastic, and tumor mammary epithelial outgrowth fragments. Next generation deep sequencing was used to determine if mtDNA “*de novo*” SNP variants are conserved during serial transplantation of both normal and neoplastic mammary clones. Our results support the conclusion that mtDNA “*de novo*” SNP variants are selected for and maintained during serial passaging of clonal phenotypically heterogeneous normal cellular populations; neoplastic cellular populations; metastatic clonal cellular populations and in individual tumor transplants, grown from the original metastatic tumor. In one case, a mammary tumor arising from a single cell, within a clonal hyperplastic outgrowth, contained only mtDNA copies, harboring a deleterious “*de novo*” SNP variant, suggesting that only one mtDNA template may act as a template for all mtDNA copies regardless of cell phenotype. This process has been attributed to “heteroplasmic-shifting”. A process that is thought to result from selective pressure and may be responsible for pathogenic mutated mtDNA copies becoming homogeneous in clonal dominant oncogenic tissues.

## Introduction

Since the advent of rapid sequencing technology, it has become more advantageous for researchers to investigate evolutionary history of individual tumors. Assessing tumor clonality in heterogenous populations requires stable reliable biomarkers that remains conserved throughout clonal expansion. Early methods for assessing tumor clonality used X chromosome-linked studies such as: glucose 6 phosphate dehydrogenase (G6PD) [1]; phosphoglycerate kinase (PGK) [2]; and a human androgen receptor (HUMARA) [3]. Interestingly, relying on the random inactivation of the X chromosome, process known as lyonization, only makes this method most accurate and useful for assessment in females. Another method analyzed and compared loss of heterozygosity (LOH) in microsatellite regions of chromosomes [4]. Currently, analysis of mitochondrial DNA, as another molecular genomic marker, has piqued interest. Several earlier studies focused on the D Loop (displacement loop) in the control region of the mtDNA. Specifically identifying a mononucleotide Cytosine repeat, in the hypervariable 2 (HVR2) region, known as D310. Utilizing the D310 repeat has shown promise in assessing clonality in a myriad of cancers: lung cancers [5], head and neck cancers [5], and several other solid tumors. However, due to the D310 mutation localized in the hypervariable region, where increased variation persist, effective use of the variation as a stable biomarker remains inconclusive.

Our study analyzes mtDNA sequence conservation or alteration in the clonal progeny of a single cell, which was identified by retroviral genomic insertions and followed through various stages of carcinogenic progression. The CzechII mouse represents a unique model system for determining the role of the MMTV in promoting mammary tumorigenesis [6]. This is manifest in the absence of MMTV sequences in the CzechII mouse genomic DNA. The CzechII colony is the only mouse line inbred or otherwise that is negative for MMTV DNA sequences in its germline DNA. As a result, all MMTV-DNA insertion events can be mapped within the mammary somatic genome whether oncogenic transformation results or not. Thus, it was possible to include non-tumorigenic “normal” MMTV-infected mammary clones, as well as premalignant and malignant mammary clones in our study.

It has been established that both normal and hyperplastic outgrowths are stable clonal populations by retroviral (MMTV)-marking [6–8]. Our present study utilizes next generation sequencing (NGS) on mtDNA extracted from serially transplanted, clonally derived, non-tumorigenic mammary outgrowths [7, 8] clonal mammary epithelial hyperplasia, and mammary epithelial tumors arising within these populations [6].

## Results

### Isolation of mitochondrial DNA from snap frozen tissue, utilizing Qiagen Qproteome and DNeasy isolation kits

**Figure 1:**
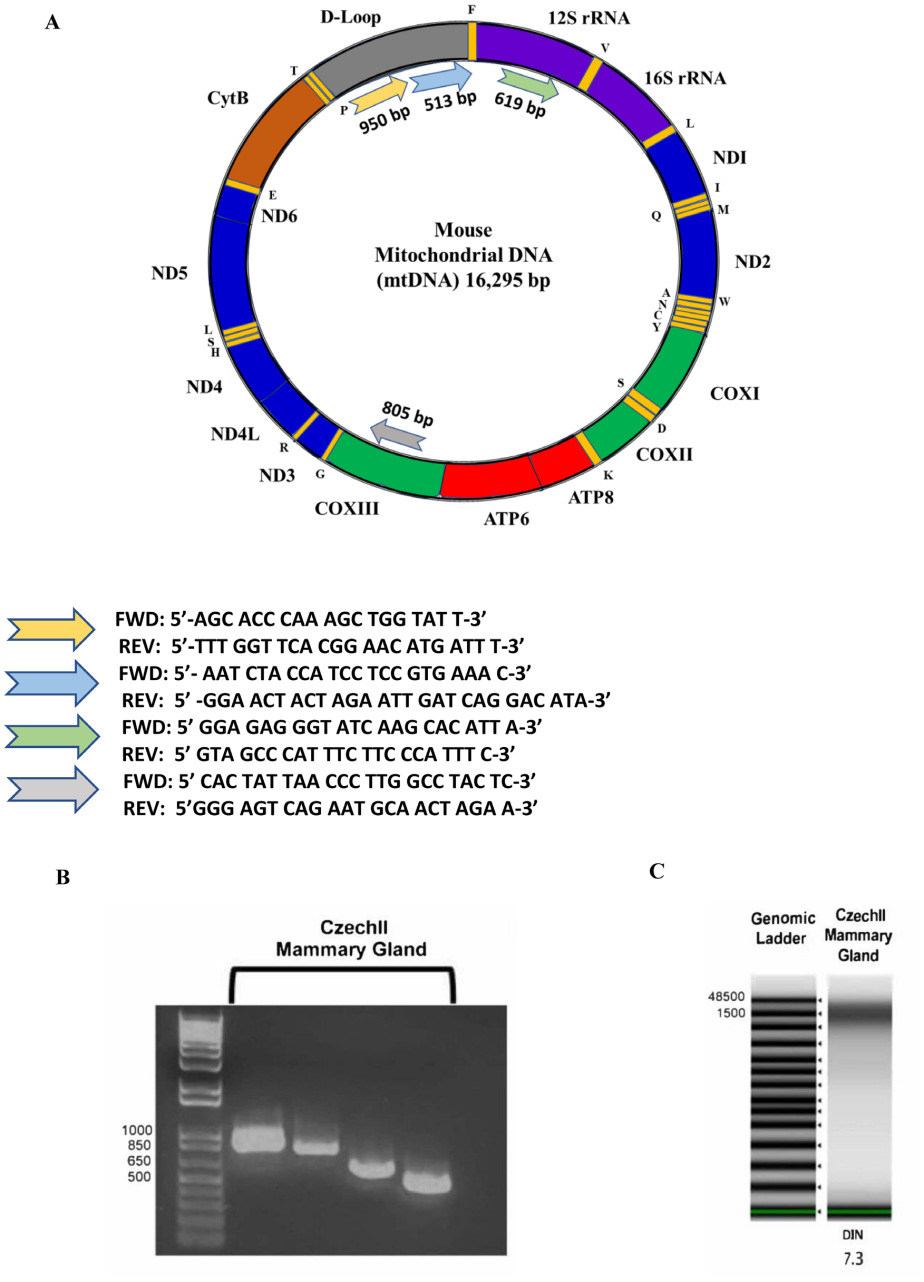
Mouse Mitochondrial Genome. PCR primers were designed reference mtDNA sequence of Blab/C mouse strain. PCR primers were designed to see if the full mtDNA sequence was present (**A**). Mouse Mitochondrial Gel Electrophoresis. 1 kb plus ladder (Lane 1) and mtDNA PCR products of a non-tumorigenic mammary control (Lane 2–5), bands align at the expected lengths of 950 bp, 805 bp, 619 bp, and 513 bp with the exception of the first and third band of the mammary control (**B**). Mitochondrial Genomic ScreenTape assay. Agilent 2200 TapeStation quantifies DNA and detects integrity of DNA by using a DNA integrity number (DIN). Czech mammary epithelial tumor mtDNA was extracted using two different methods. DNA Ladder (Lane 1). MtDNA extracted using Qiagen QIAPrep Spin Miniprep Kit (Lanes 2–6 and 9). MtDNA extracted using Qiagen DNeasy Blood and Tissue Kit (Lanes 7–8). DNeasy methodology successfully extracted intact mtDNA from mammary epithelial tumors (**C**).

A cartoon schematic shows our methods of utilizing two Qiagen isolation kits, Qproteome and DNeasy, to isolate intact mitochondria and mitochondrial DNA, using snap frozen starting material (Supplementary Figure 1A, 1B). After isolation of mitochondrial DNA, we validated the presence of mitochondrial DNA by PCR. Primer sets were designed to target 4 arbitrary regions in the mouse mitochondrial genome: Trn-Pro, Co3, Rnr1 and D-Loop (Figure 1A). On a 1% agarose gel, the DNA fragment patterns are at the desired base pair length: Trn-Pro (950 bp), Co3 (805 bp), Rnr1 (619 bp) and D-Loop (513 bp) (Figure 1B). To ensure mitochondrial DNA is not fragmented, we performed genomic TAPE assay to measure mitochondrial integrity. Results show mitochondrial DNA appears around 16,000 bp in length with 7.3 DIN (Figure 1C).

### Mitochondrial DNA bioinformatic analysis and phylogenetic mapping reveals CzechII mouse relationship distance from the mtDNA of 39 common mouse strains

**Figure 2:**
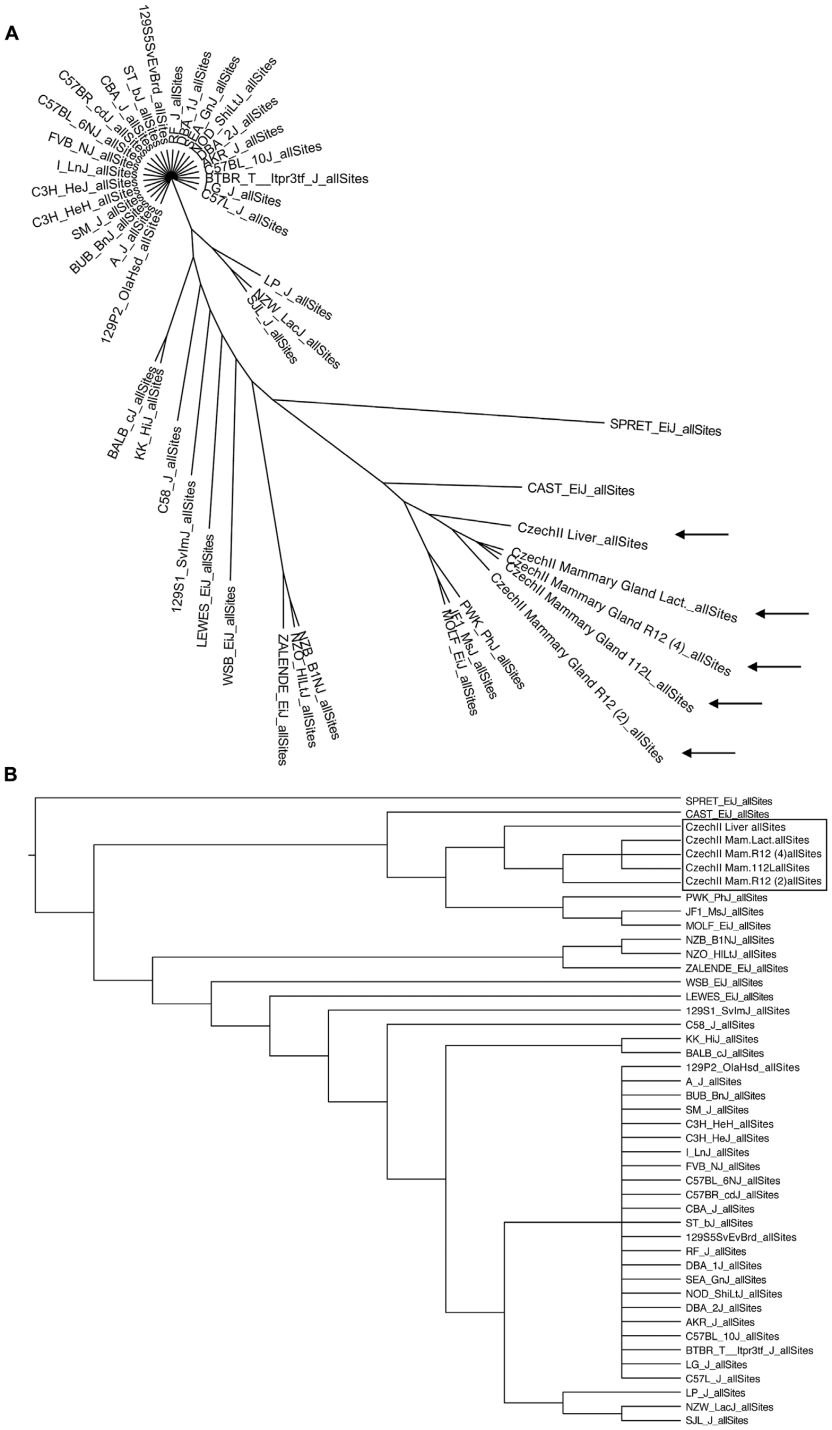
Phylogenetic mapping of CzechII mouse strain. CzechII and 39 common mouse strains phylogenetically mapped, utilizing bioinformatic software and the Sanger Mouse Genome Project (**A**, **B**).

Bioinformatic analysis, of CzechII mtDNA, enabled phylogenetic mapping. Comparison and genotyping of CzechII mouse genome and 39 common mice strains (i. e. C57 black and Balb/C), was performed utilizing the Sanger Mouse Genome Project. Phylogenetic network construction revealed, via mtDNA analysis and genotyping, glaring separation between CzechII mtDNA and that of common mouse strains (Figure 2A, 2B).

### Next generation deep sequencing identifies 2 “*de-novo*” SNP variants conserved in R12 MT1 tumor transplants and lung metastasis

Next Generation sequencing, with a mitochondrial genome coverage of ≥50× at ≈98% (Supplementary Table 1), was performed on normal mammary generational outgrowths of R12 and L12. SNP variant calling analysis identified CzechII mt-SNPs which were filtered to reveal minor somatic SNP variants, throughout CzechII normal mammary outgrowths. R12 normal outgrowths revealed no significant change in the mitochondrial genome compared to the controls, mtDNA isolated from snap frozen liver and lactating mammary gland. The same conservation pattern, i. e. wild type, was observed in the mitochondria extracted and sequenced from multiple (*n* = 5) L12 second transplant generation lactating outgrowths. In agreement with the results obtained with mtDNA isolated from lactating R12 serial transplants (Data not shown).

**Figure 3:**
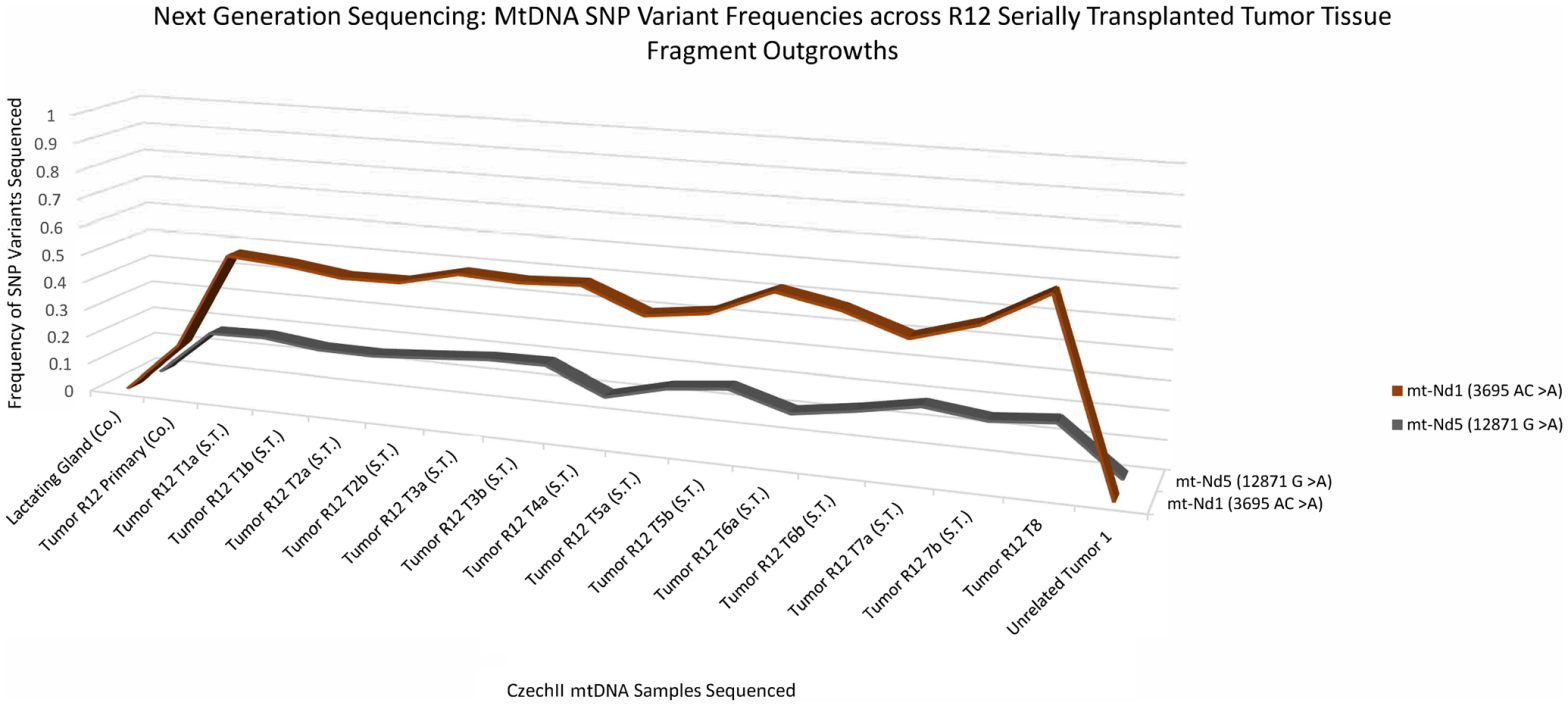
CzechII mammary R12 tumor mtDNA SNP variant calling via Next Generation sequencing. Next generation sequencing was performed on R12 mammary tumor and the tumors from 7 serially transplanted CzechII mammary R12 tumor fragments, SNP variant calling was performed to analyze common somatic SNPs that were conserved across R12 tumor fragments in comparison to CzechII Lactating mammary gland, negative control and CzechII Primary R12, positive control. Samples ran in duplicates.

**Figure 4:**
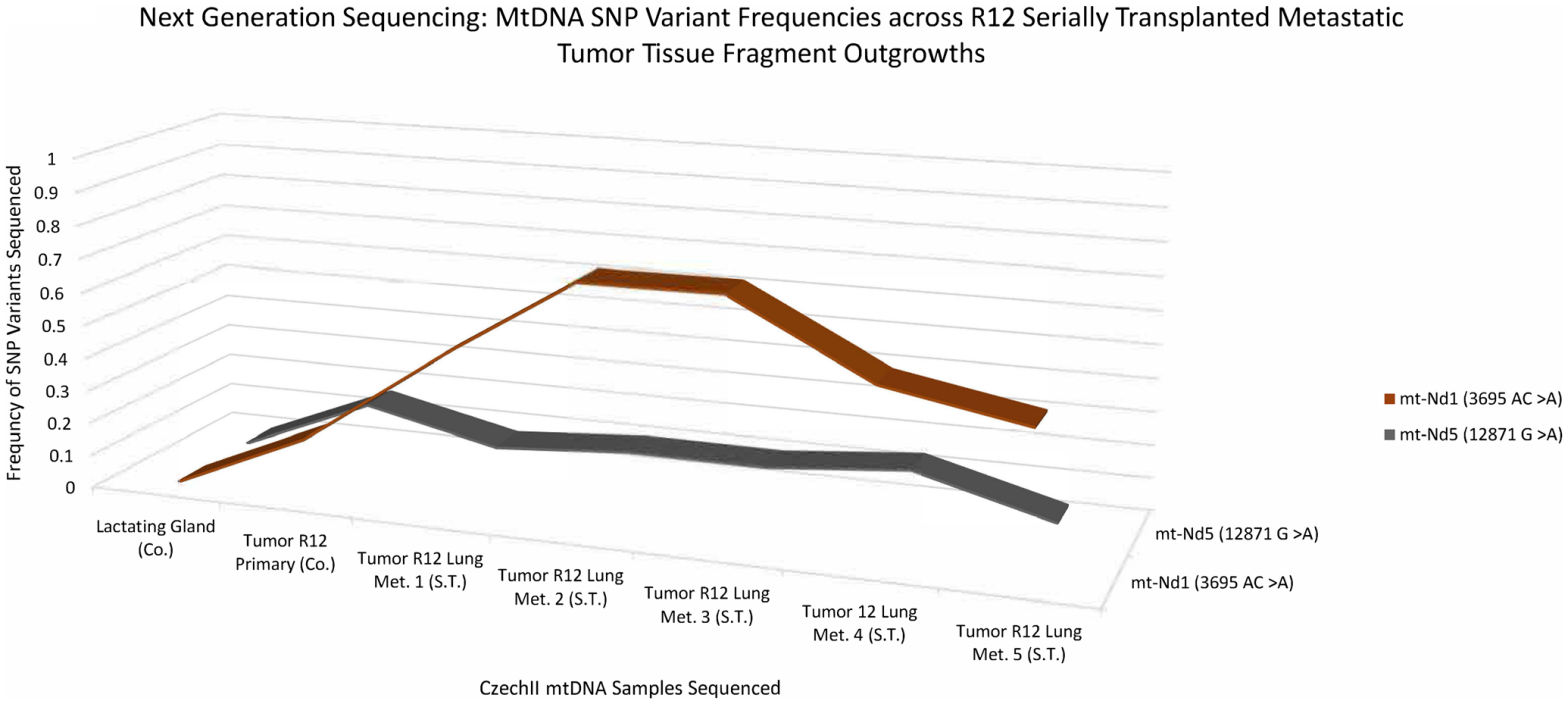
CzechII mammary R12 metastatic tumor mtDNA SNP variant calling via Next Generation sequencing. Next generation sequencing was performed on R12 mammary tumor and 5 CzechII mammary R12 serially transplanted metastatic tumor fragments from R12 Tumor, SNP variant calling was performed to analyze common somatic SNPs that were conserved across R12 metastatic tumor fragments in comparison to CzechII Lactating mammary gland, negative control and CzechII Primary R12, positive control.

A 3D line graph (Figure 3) shows the appearance and conservation of two “*de-novo*” SNP variants, mt-ND1 3695 AC>A and mt-ND5 12871 G>A, which were not present in normal R12 or controls but later appeared in subsequent R12 tumor clone transplants. Next Generation sequencing data reveals a mt-ND1 SNP variant, 3695 AC>A and an mt-ND5 SNP variant, 12871 G>A, in a primary R12 tumor in the near equivalent of 17%. In R12 tumor transplants the mt-ND1 SNP variant, 3695 AC>A, increased in frequency to approximately 45% to 55% and remained conserved throughout samples. Alternatively, the mt ND5 SNP variant, 12871 G>A, remained at a frequency of 17%, in all the tumor transplants. Mt-ND1 SNP variants, 3695 AC>A and mt-ND5 SNP variant, 12871 G>A, were not found in mt-DNA isolated from an unrelated CzechII mammary tumor arising in the same mouse. The R12 lung metastatic tumor transplants, show a similar pattern of increased frequency for the mt-ND1 SNP variant, 3695 AC>A. R12 lung metastasis, compared to control, the mt- ND1 SNP increases to approximately 45% to 75% and was conserved throughout samples. Similar to the preceding tumor transplants, the mt ND5 SNP variant, 12871 G>A remained conserved at the same frequency (17%) (Figure 4).

*In silico* analysis of, mt ND1 (3695 AC>A) and mt ND5 (12871 G>A) in CzechII R12 tumor transplants, investigated SNP variant consequence; SNP variant impact; protein position; amino acid changes; and SIFT prediction of the SNP variant impact on amino acid changes which may affect protein function. The conserved mt ND1 SNP variant, 3695 AC>A, was shown to be a deleterious, frameshift mutation and had high impact on DNA sequence that leads to a truncated or non-functional ND1 protein. Mt ND5 SNP variant, 12871 G>A, was shown to be a tolerated missense mutation and was predicted to have a moderate impact on DNA sequence that may impact ND5 protein function (Table 1).

**Table 1:** SNP Variants of Interest (VOI) identified by next generation sequencing in R12 and CZN5

Chromosome	Tumor Sample ID	Gene	Variant Position	Reference/ Alternative	Variant Consequence	Variant Impact	Protein Position	Amino Acid Change	SIFT
Mitochondria	R12 (MT1)	mt-Nd5	12871	G/A	Missense	Medium	377	S/N	Tolerated
Mitochondria	**R12** **(MT1)**	**mt-Nd1**	**3695**	**AC/A**	**Frameshift**	**High**	**316**	**P/X**	**Deleterious**
Mitochondria	CZN5 (MT2)	mt-Co1	1017	G/T, A	Upstream Gene	Modifier	—	—	—
Mitochondria	**CZN5** **(MT1)**	**mt-Nd1**	**3274**	**T/TAC**	**Frameshift**	**High**	**175**	**L/LX**	**Deleterious**

Note: SIFT (Sorting Intolerant from Tolerant) score predicts impact of amino acid substitutions based on the degree of conservation in sequence alignments derived from closely related sequences. Scores <0.05 are considered deleterious.

### Next generation deep sequencing identifies 2 “*de-novo*” SNPs conserved in CZN5 hyperplasia to tumorigenesis

**Figure 5:**
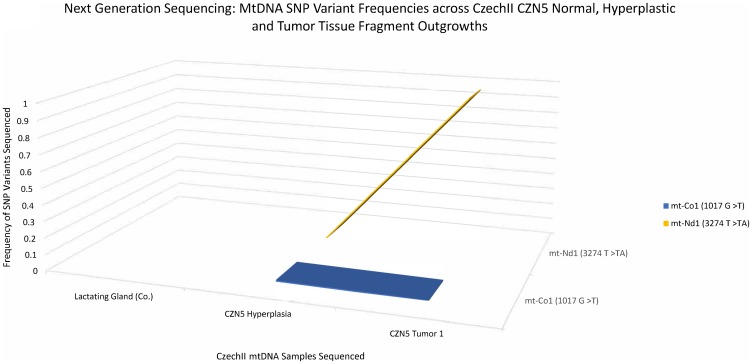
CzechII mammary CZN5 tumor 1 mtDNA SNP variant calling via Next Generation sequencing. Next Generation sequencing was performed on CZN5 tumor 1 that arose from a CzechII CZN5 hyperplasia. SNP variant calling was performed to analyze common somatic SNPs that were conserved across CZN5 hyperplasia and tumor outgrowth, in comparison to CzechII lactating mammary gland control.

MtDNA was isolated from CZN5, a premalignant CzechII clonally derived outgrowth line. Next generation deep sequencing, with a mitochondrial genome coverage of ≥50× at ≈98% (Supplementary Table 1), was performed to analyze somatic SNP variants that were conserved in the CZN5 hyperplasia and the mammary tumors that stochastically developed in this population during its serial passage. A 3D line graph (Figure 5) illustrates two “*de-novo*” conserved SNP variants, mt-ND1 (3274 T>TA) and mt-CO1 (1017 G>T), develop in CZN5 mammary tumors, which did not appear in the lactating mammary gland control. Mt-ND1 SNP variant (3274 T>TA), appeared at 0% frequency in the antecedent CZN5 hyperplasia but became fixed at 100%, in the succeeding CZN5 tumor 2. The second SNP variant, mt- Cox1 (1017 G>T), appeared to be at 0% frequency, which showed conservation of frequency throughout CZN5 hyperplasia and CZN5 tumor 1.

**Figure 6:**
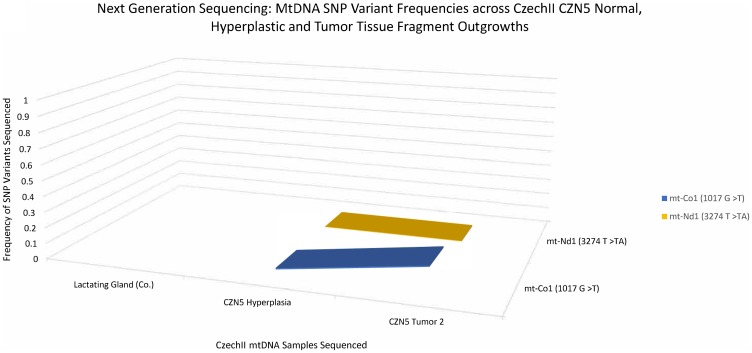
CzechII mammary CZN5 tumor 2 mtDNA SNP variant calling via Next Generation sequencing. Next Generation sequencing was performed on CZN5 tumor 2 that arose from a CzechII CZN5 hyperplasia. SNP variant calling was performed to analyze common somatic SNPs that were conserved across CZN5 hyperplasia and tumor outgrowth, in comparison to CzechII lactating mammary gland control.

The two SNP variants were also identified in CZN5 mammary tumors. However, the mt-ND1 SNP variant, (3274 T>TA), displayed 0% frequency, in the antecedent hyperplasia and remained consistent throughout tumor development. Interestingly, mt-CO1 SNP variant (1017 G>T) appeared at 0.8% frequency, in CZN5 hyperplasia and later increased to 14% frequency, in CZN5 tumor 2 (Figure 6).

*In silico* analysis of mt-ND1 (3274 T>TA) and mt-Co1 (1017 G>T) in the CzechII CZN5 tumor 1 samples, identified SNP variant consequence; SNP variant impact; protein position; amino acid changes; and SIFT prediction of SNP variant impact on amino acid changes which may affect protein function. The conserved mt ND1 (3274 T>TA) was shown to be a deleterious, frameshift mutation and had a high impact on DNA sequence that may lead to engendering a non-functional ND1 protein. Mt-Co1 (1017 G>T) was shown to be a modifier due to its position outside the coding region, no functional data was predicted for this SNP variant (Table 1).

### R12 and CZN5 tumors, harboring “*de-novo*” SNP variant (3695 AC>A) and (3274 T>TA) respectively, reveals significant decrease in mt-ND1 gene expression

**Figure 7:**
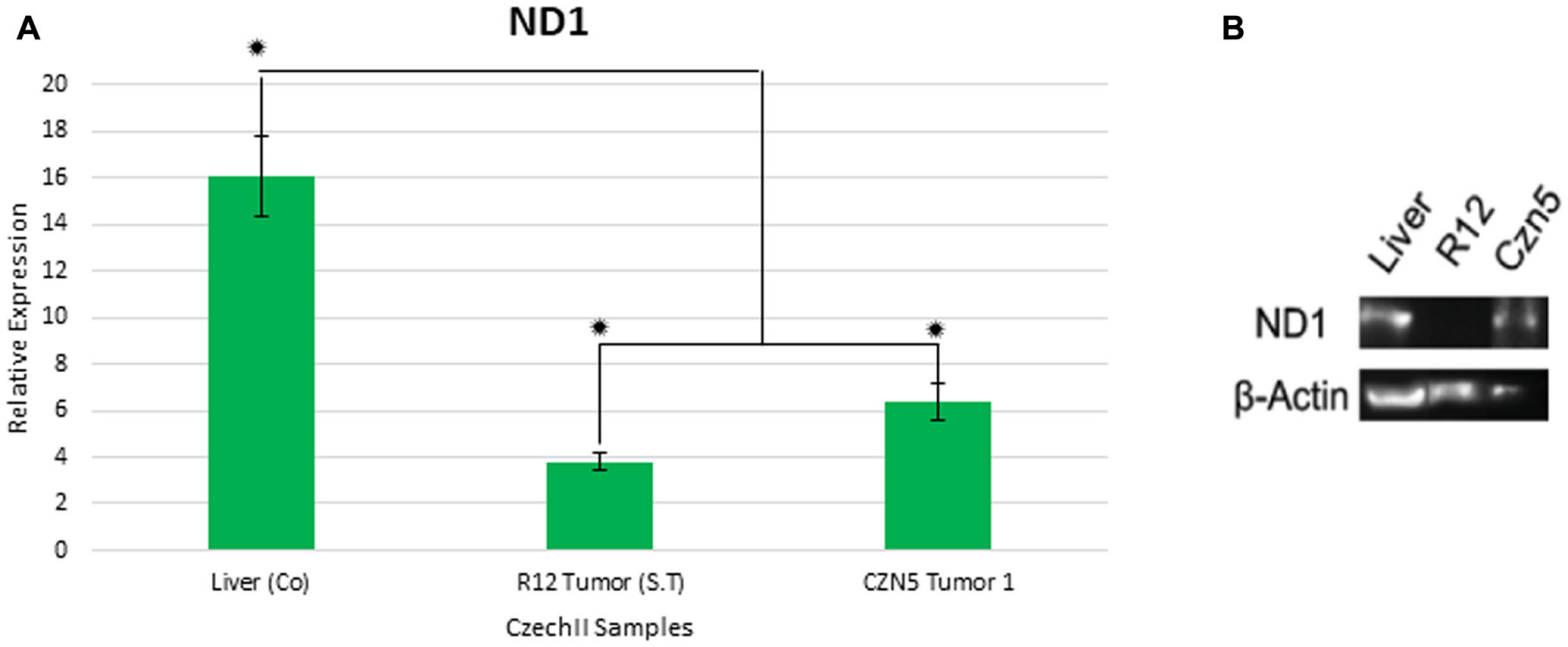
Gene expression of mt-Nd1 in CzechII tumor samples. Mt-Nd1 expression was measured by performing digital droplet polymerase chain reaction (ddPCR) on CzechII R12 Tumor and CZN5 tumor 1. Mt-ATP5f1 was used as endogenous control. CzechII R12 tumor 1 and CZN5 Tumor 1 Nd1 relative gene expression was compared to CzechII liver control. Statistical analysis was performed using ANOVA and Post-Hoc student *t*-test, Bonferroni correction *P* < .05. Samples were run in triplicates. Protein expression of mt-Nd1 in Czech tumor samples. Mt-ND1 protein expression was measured by performing a western blot on CzechII R12 Tumor and CZN5 tumor 1 (**A**). β-actin was used as endogenous control. CzechII R12 tumor 1 and CZN5 tumor 1 ND1 protein expression was compared to CzechII liver (**B**).

Next generation sequencing identified two different deleterious “*de novo*” mt ND1 SNP variants, in R12 and CZN5 tumors. We investigated the deleterious effects of the SNPs, in ND1, due to possibly elucidating the potential need, of the mutation, in mammary tumorigenesis. *In silico* analysis revealed these SNP variants to be deleterious frameshift mutations, that highly impact mt ND1 sequence, leading to decrease in mt ND1 gene expression. To validate the predicted impact of SNP variants on, mt ND1 sequence, ddPCR analysis was performed on the R12 and CZN5 tumors harboring the two “*de novo*” SNP variants. Statistical analysis of ddPCR mt ND1 gene expression results revealed a 2.5-fold decrease in CZN5 tumor 1 and a 4-fold decrease in CzechII R12, compared to CzechII Liver (Control), Bonferroni Corrected *p* < .05 (Figure 7A).

### R12 and CZN5 tumors, harboring “*de-novo*” SNP variant (3695 AC>A) and (3274 T>TA) respectively, reveal a significant decrease in mt-ND1 protein expression

Next generation sequencing identified two different “*de novo*” mt ND1 SNP mutations in R12 and CZN5 tumors. *In silico* analysis revealed these SNP mutations to be deleterious frameshift mutations that highly impacted the mt ND1 sequence. To validate the impact of the mutations on ND1 protein function, a western blot was performed on R12 and CZN5 tumors harboring the two “*de novo*” SNP mutations. β-actin was used as an endogenous control. Lane 1 shows control, CzechII liver, having normal expression of ND1. However, compared to R12 tumor, absence of fluorescent band indicates complete ablation of ND1 protein expression. In addition, attenuation of band fluorescence in CZN5 indicates normal ND1 protein function being significantly reduced (Figure 7B).

## Discussion

The potential of mtDNA being used as a molecular biomarker for breast cancer, like other malignant tumors, to determine cancer clonality, has been widely investigated [9–14]. The attractiveness of mtDNA, as a clonal molecular biomarker, may derive from its forensic applications [15], uses in phylogenetic and evolution mapping [16]. The reason for its diverse use of application is due, in part, to mitochondrial evolution, which produced sequences that remain tightly conserved among species (e. g. 12S mt-rDNA) and between species (e. g. 16S mt-rDNA and mt-Cyt b) [17]. In addition, mtDNA possess highly varied sequences in the control region known as hypervariable region 1 and 2, which varies greatly between species. Several early studies investigated the control and hypervariable regions probably because of the higher frequency of non-functional mutations that did not impact the coding region, resulting in no selective pressure to degrade the mutated mtDNA [18]. However, in doing so, the mechanism that may underlie “selective pressure “and mitochondrial homoplasmy, to engender sub clonal dominant clones, would be ignored.

To test whether mtDNA harbored somatic “*de novo*” SNP variants, that could be detected in clonal populations during clonal expansion, we isolated and extracted mtDNA, to perform next generation deep sequencing and bioinformatic variant calling.

An important aspect of our current analyses of mtDNA is that all tissues were clonal. In other words, R12 and L12, both apparently normal mammary outgrowths, were shown to be derived from a single cell by Southern blot analyses for MMTV DNA insertions, which are known to be random [7, 8]. All the MMTV-induced mammary premalignant outgrowths (HOG) lines were also shown to be derived from a single antecedent by virtue of Southern blots showing identical MMTV DNA insertions at each passage [6]. The mammary tumors arising within the premalignant outgrowths arise from single cells and share all the MMTV DNA insertions found in each individual premalignant outgrowth line [19]. Therefore, all of the mammary mtDNAs, except the non-clonal lactating mammary gland and liver, are derived from the progeny of a single cellular antecedent. This is important because, in effect, mtDNA in the progeny from a single cell is analyzed from normality through progression to full malignancy.

In CZN5 hyperplasia (HOG), SNP variant mt-ND1 (3274 T>TA) was undetected. However, in the tumor antecedent, CZN5 tumor 1, the mt-ND1 (3274 T>TA) mutation frequency rose to 100% demonstrating that all mtDNA copies tested by NGS, from this tumor, possessed the mtND1(3274 T>TA) SNP variant in the same position in the mitochondrial coding region. Coller and colleagues [20] showed, through computer simulation, that mtDNA mutations in a tumor progenitor cell could lead to a homoplasmic state in human tumors by clonal cell proliferation.

Investigating the impact of mtND1(3274 T>TA) on ND1 function, further elucidates the potential need of the mutation, in CZN5 hyperplasia, leading to tumorigenesis. Our bioinformatic result suggests that ND1 (3274 T>TA) is a frameshift mutation that generates a premature stop site in the mtDNA coding sequence for ND1. Digital Droplet PCR (ddPCR) confirmed a 2.5 decrease in ND1 mRNA expression compared to its control, CzechII liver mitochondria. This was verified by western blot showing attenuation of the band intensity of ND1 protein expression. ND1 dysfunction, in complex 1 of the electron transport chain (ETC) of the mitochondria, has been documented to be associated with tumor growth [21–23]. It’s important to recognize that this tumor population developed from a single “tumor progenitor” cell in the CZN5 hyperplastic population. Therefore, although ND1 (3274 T>TA) may be important in CZN5 tumor 1 through positive selection, it may have arisen randomly during clonal expansion due to “heteroplasmic shifting”.

The CZN5 hyperplasia tested, harbored a SNP mutation in its mtDNA, Cox1 (1017 G>T), which was also detected at 0.7%. However, in CZN5 tumor 2, that SNP frequency increased to 14.0%. This is indicative of “heteroplasmic shifting”. Conversely, since the tumor is comprised largely of epithelium whereas the HOG is a mixture of stroma and epithelium, it may simply reflect the increased mtDNA contribution of the epithelium. Additional CZN5-derived mammary tumor mtDNAs, that were analyzed, were devoid of any SNP found in mtDNA from the control CzechII tissues (data not shown). This supports randomness in clonal expansion, evident in each “tumor progenitor” cell within the premalignant population.

The normal R12 outgrowth gave rise to a single tumor (R12 MT1). The mtDNA from this tumor possessed two “*de novo*” SNP variants, ND1 (3695 AC>A) and ND5 (12781 G>A). Each was present in approximately 17% of the mtDNA sequences analyzed in the primary tumor. However, in the R12 tumor transplants and lung metastases, the ND1 SNP variant increased dramatically compared to the ND5 SNP variant, which remains approximately at the same frequency as in MT1. Therefore, these SNPs are in separate mtDNA genomes. Our bioinformatic analysis results indicate that ND1 (3695 AC>A) is a frameshift mutation that introduces a premature stop site in the mtDNA coding sequence. The SNP variant in ND5 was predicted to be a tolerable missense mutation, which may not impact protein function but may be involved in tumor progression [24]. A clear explanation for two separate mtDNA mutations in a clonally derived tumor is challenging unless one considers that one SNP preceded the second during the clonal expansion of the tumor population. This could result in two independent tumor sub clones each characterized by different mtDNA SNPs, i. e., ND1 (3695 AC>A) and ND5 (12781 G>A). In the lung metastases and tumor transplants, the sub clone bearing the ND1 SNP became the larger contributor of mtDNA copies. Whether this is due to a selective advantage for growth in distal sites is unclear. However, ND1 (3695 AC>A) is a SNP of interest due to its classification as a frameshift mutation and deleterious impact on ND1 protein function. Interestingly, which may suggest a selective advantage for cells harboring mutations later proliferating in sub clonal dominant tumor.

Digital Droplet PCR (ddPCR) showed a 4.2-fold decrease in ND1 mRNA compared to its control, CzechII liver mtND1 mRNA. The deleterious impact of ND1 (3695 AC>A) on the mitochondrial sequence, leading to dysfunction in the electron transport chain (ETC), may be important in this sub clone for its proliferation in distal sites.

There are currently three leading theories of the mtDNA inheritance mechanisms: (1) variation in heteroplasmy is due to an unequal segregation of mtDNA during cell division, (2) variation in heteroplasmy is due to an unequal segregation of mtDNA nucleoids during cell division and (3) variation in heteroplasmy is due to the selective replication of a specific sub-population of mtDNA [25]. A clear and cogent argument from our results states that mitochondrial DNA synthesis must be very precise because one seldom detects a specific SNP in their DNA sequence. Only the tumors arising within clonal outgrowths, which are clones themselves, possess “*de Novo*” SNPs. These are detected at identical positions in their mtDNA sequence and the nucleotide changed is always the same. This argues that for these mtDNAs there is extreme fidelity in their replication. There are two distinct SNPs in MT1 of the R12 outgrowth mtDNAs, one is found in the coding region of the ND1 gene and another in the coding region for subunit ND5. This suggests the presence of two distinct cellular sub clones within the MTI tumor containing mutated mtDNA. One sub clone with only the ND1-SNP in its mitochondrial DNA and one sub clone with the ND5-SNP variation. The increase in the frequency of ND1-SNP in the mtDNAs isolated from the lung metastases and from the tumor transplants of MT1 can be explained by the selective increase in the 1st sub clone. The best example of the fidelity of mtDNA replication was discovered in CZN5 MT1, where all the analyzed mtDNA sequences contained a SNP at the identical position in the ND1 coding gene. These data suggest that a single mutated mtDNA genome acts as the template for all others in any given clone during its expansion. We realize that this hypothesis for mtDNA replication and inheritance is contrary to the generally accepted view. However, our data is best explained by this concept and is supported by the observation that “normal”, premalignant and malignant cell populations may develop from a single retrovirally-marked antecedent.

## Materials and Methods

### Mice

CzechII mice infected with mouse mammary tumor virus (MMTV) [26] were used as donors and hosts of the mammary epithelial transplants. The mice were held in a closed colony, maintained on a 12-h light/dark cycle under controlled temperature and humidity, and were given laboratory chow supplemented with birdseed and water ad libitum. Bedding was hardwood chip and was autoclaved prior to use. All animal care and treatment were conducted strictly according to the rules and procedures defined by the USPHS and the NIH and were approved by the NCI Animal Care and Use Committee.

### Mammary tissue transplantation

The surgical procedures for clearing the mammary epithelium from the inguinal fat pads of 3-week-old female mice and the method of implanting either tissue fragments or cell suspensions have been described in detail in earlier publications [27–31]. The surgical procedures required to remove the host epithelium from the fat pads were performed immediately prior to insertion of the transplant or inoculation of cultured cells. The development and characterization of the clonally derived outgrowths and their serial transplantation into epithelium-cleared mammary fat pads has been described in detail [7, 8]. The implanted glands as well as intact host glands were taken 1 day postpartum.

### Intact mitochondrial isolation

All tissue samples (*N* = 34) were snap frozen in liquid N2 and stored at –80C. A portion of the frozen tissue was thawed on ice and washed using 1 mL 0.9% (w/v) sodium chloride solution. If necessary, the tissue was cut into ~2 mm^3^ pieces and placed into a 2 mL reaction tubes. Lysis Buffer (500 µL, supplemented with Protease Inhibitor Solution) was added to each reaction tube. Dissertator rotor-stator homogenizer set at the lowest speed for 10 s, was used to homogenize tissue sample. After disruption the solution was incubated on an end-over-end shaker for 10 min at 4°C. Homogenate was centrifuged at 1000 × g for 10 min at 4°C. Supernatant was carefully removed. The pellet was resuspended in 1.5 mL ice-cold Disruption Buffer. Lysate was drawn into a 1.0 cc syringe equipped with a 25-gauge needle and ejected with one stroke, 10 times. Lysate was then centrifuged at 1000 × g for 10 min at 4°C. The supernatant was carefully transferred to a clean 1.5 mL tube. Supernatants from each extraction were combined. Supernatant (s) were centrifuged at 6000 × g for 10 min at 4°C. Mitochondrial pellet was washed with 1 mL Mitochondria Storage Buffer. This solution was centrifuged at 6000 × g for 20 min at 4°C (Qproteome Mitochondria Isolation Kit, Qiagen).

### Mitochondrial DNA preparation

Qiagen DNeasy kit was used to extract mtDNA. This was done according to the manufacturer’s protocol. Proteinase K (20 µL) and PBS (200 µL) were added to the mtDNA pellet for resuspension.

### Genomic DNA screentape assay

Quantification, Sizing and Integrity Analysis of CzechII mtDNA using the Agilent 4200 TapeStation system (G2991AA), Genomic DNA ScreenTape (5067-5365) and Genomic DNA Reagents (5067-5366) were obtained from Agilent Technologies. The extracted mtDNA was analyzed using the Genomic DNA ScreenTape assay. The samples were prepared by mixing 1 µL of gDNA sample with 10 µL of Genomic DNA Sample buffer. A 3 µL amount of Genomic DNA Ladder was placed in the first tube of an 8-way strip followed by the samples. The prepared strip was vortexed on high speed for 5 seconds, centrifuged and placed in the 2200 TapeStation instrument. The samples were analyzed as triplicates for each individual extracted sample.

### Next generation sequencing of CzechII mtDNA from serially transplanted normal, tumor, and metastatic CzechII mammary tissue

A DNA library was prepared using the Nextera XT library preparation kit (Illumina). The concentrations of the indexed libraries were analyzed on the Agilent 4200 TapeStation (Agilent Technologies) using the D1000 Kit (Agilent Technologies). For CzechII L12 serially transplanted normal lactating mammary tissue mtDNA, equimolar amounts of the 5 indexed libraries were pooled to obtain a 4 nM library mixture. CzechII R12 serially transplanted normal mammary tissue mtDNA, equimolar amounts of the 4 indexed libraries were pooled to obtain a 4 nM library mixture. CzechII R12 serially transplanted tumor mammary tissue mtDNA, equimolar amounts of the 15 indexed libraries were pooled to obtain a 4 nM library mixture. CzechII R12 serially transplanted metastatic mammary tissue mtDNA, equimolar amounts of the 5 indexed libraries were pooled to obtain a 4 nM library mixture After denaturing, and further diluting, the final 1.3 pM library was loaded into an Illumina cartridge. Sequencing was performed using the Illumina NextSeq 500/550 High Output Kit v2 (300 Cycles) on the Illumina NextSeq 500 instrument following the manufacturer’s instructions (Illumina). For CzechII CZN5 serially transplanted hyperplastic and tumor mammary tissue mtDNA, equimolar amounts of the 5 indexed libraries were pooled to obtain a 4 nM library mixture. After denaturing, and further diluting, the final 1.3 pM library was loaded into an Illumina cartridge. Sequencing was performed using the Illumina NextSeq 500/550 High Output Kit v2 (300 Cycles) on the Illumina NextSeq 500 instrument following the manufacturer’s instructions (Illumina).

### MtDNA SNP variant calling and analysis

All NGS data processing was done using our in-house developed pipeline (https://github.com/CCBR/Pipeliner), with slight modifications to accommodate mtDNA SNP variant calling. Short read data was trimmed for the presence of adaptors and low quality using Trimmomatic v0.36 48 [32] and the following parameter settings: Leading:10; Trailing:10; Sliding window:4:20; Minlen:20. Reads were then mapped to the mm10 reference genome using BWA-mem v0.7.15 with default parameter settings (https://arxiv.org/abs/1303.3997). The resulting BAM files were sorted using SAMtools v1.317 and PCR duplicates were marked using Picard v2.1.1 (https://broadinstitute.github.io/picard/). Realignment around INDELs and base recalibration was performed using the Genome Analysis Toolkit v.3.5 (GATK, Broad Institute, Cambridge, MA, USA), following the GATK Best Practices [33, 34]. For somatic SNP variant and INDEL detection, we used MuTect2 (https://software.broadinstitute.org/gatk/documentation/tooldocs/current/org_broadinstitute_gatk_tools_walkers_cancer_m2_MuTect2.php) with the ploidy flag set to 1. SNP variant calling was run paired, with each sample genotyped jointly with the CzechII liver mtDNA control sample. Resulting candidate somatic SNP variant calls were then hard-filtered using the following criteria: 1) for SNPs – Fisher’s Strand (FS) > 60.0, Stand Odds Ratio (SOR) > 3.0, Mapping Quality (MQ) < 40.0, Mapping Quality Rank Sum Test (MQRankSum) < -12.5, and ReadPosRankSum < -8.0; 2) for INDELs - FS > 200.0, SOR > 10.0, ReadPosRankSum < -20.0. All remaining SNP variants outside of the mtDNA were discarded, and mtDNA SNP variants were then annotated using VEP v94 [35].

### Bioinformatic phylogenetic network of CzechII mouse strain with other known mouse strains via mtDNA analysis

To examine the relationship of our CzechII mice with other known strains, a germline phylogenetic network was generated using all published mouse strain mtDNA genomes available through the Sanger Mouse Genome Project (39 total strains; https://www.sanger.ac.uk/science/data/mouse-genomes-project). To homogenize the CzechII samples with the Sanger mouse strain genomes, BAM files for each mouse strain were downloaded and reads were extracted for each genome using the bamtofastq tool in bedtools v2.27.2 (https://bedtools.readthedocs.io/en/latest/) and remapped with the above pipeline. All samples were then joint genotyped using the HaplotypeCaller from the Genome Analysis Toolkit v.3.5 (GATK, Broad Institute, Cambridge, MA, USA), and following the GATK Best Practices [33, 34]. Uncorrected genetic distances were generated using plink v1.07 [36] and a phylogenetic network was generated using phylip v3.697 (http://evolution.genetics.washington.edu/phylip.html).

### Droplet digital polymerase chain reaction (ddPCR)

Reverse transcription was performed using ABI/Thermo, high-Capacity cDNA reverse transcription Kit (Cat#4368814), according to manufacturer’s protocol. 20 uL reaction with 2 ug total RNA included: 2 ul 10× buffer, 2 ul 10× random primer, 0.8 ul 25× dNTP, 1 ul Multiscreen RT.

Copy number of the ND1 gene was determined using the QX200 Droplet Digital PCR (ddPCR) System (Bio-Rad Laboratories, Hercules, CA, USA) according to the manufacturer’s instructions. Briefly, each reaction consisted of 11 μl ddPCR Supermix for probes (no dUTP), 1.1 μl of custom mouse mt-ND1 assay (IDT; Sequence 1: 5′GCCCATTCGCGTTATTCTTTAT 3′; Sequence 2: 5′ AGTTAGTTGAGTAGAGTTCTGGTAAG 3′; Sequence 3: /56- FAM/ACGCCCTAA/Zen/C AACCATTAT CTTCCTAGG/ 3IABkFQ/), 1.1 μl of mouse (endogenous control) atp5f assay (IDT; Sequence 1: 5′ TCGCAGACAATGCTGTCC 3′; Sequence 2: 5′ GGCCCTTGTTGCCTGTAATA 3′; Sequence 3: /56-FAM/TGCTTTCT G/Zen/C TGCCGCCACA/3IABkFQ/), 7.9 μl of nuclease free water. Twenty µL of each reaction was transferred to a droplet generation cartridge and 70 µL of Droplet Generation Oil was added. Droplets generated with QX200 Droplet Generator (~40 ul) were loaded into a clean 96-well PCR plate and the plate was sealed with foil seal using BioRad pierce-able foil. PCR amplification was performed in a Bio Rad T100 thermal cycler, with the following conditions: 10 minutes at 95°C, 40 cycles of 94°C for 30 seconds and 60°C for 60 seconds with ramp rate of ~2°C/second followed by 98°C for 10minutes with ramp rate of ~1°C/second. Droplets were read in a QX200 Droplet Reader and analysis was performed using QuantaSoft 1.5 (Bio-Rad Laboratories). Negative control (water) and positive control (Czech Liver), which is known not to have deleterious mt-ND1 (3695 AC >A) or (3274 T >TA), were included in the run. Study samples were analyzed in triplicates.

### Statistical analysis

Data was presented as mean ± standard deviation (SD). We performed ANOVA to compare the difference in mt-ND1 gene copy number between Czech Liver, R12 Tumor and CZN5 Tumor 1. Post-hoc Bonferroni adjustment was applied for ANOVA analysis. Paired Student’s *t*-test was used to determine the difference in mt-ND1 gene copy number between ddPCR in Czech Liver, R12 Tumor and CZN5 Tumor 1, respectively. A *p*-value of <0.05, after Bonferroni correction, was considered statistically significant. Statistical analyses were conducted using Microsoft Excel version 16.25.

### Western blot analysis

For western blot analysis, total protein was isolated using the AllPrep Kit (Qiagen) and separated on 4–20% SDS-polyacrylamide gel electrophoresis before transferring to polyvinylidene difluoride membranes (Bio-Rad). The primary antibodies were recombinant MT-ND1 (Abcam, 1:1000 dilution), and actin (Santa Cruz, 1:200). The secondary antibody was a horseradish peroxidase-labeled donkey anti-rabbit IgG or anti-donkey IgG (Abcam, 1:2000 dilution). Imaging was done on the G: Box Mini using the SYNFEMTO Chemiluminescent Substrate (Syngene).

### Summary statement

MtDNA sequence is conserved in heterogenous populations derived from single cell during clonal expansion. This suggests that a single mtDNA copy may act as a template for others within a clonal cell population and “heteroplasmic shifting” may act as a selective pressure for certain mtDNA mutations that produce a metabolic advantage for individual clonal dominant mammary tumors.

## Conclusions

We conclude that mtDNA sequences are conserved during clonal expansion and may be selected via “heteroplasmic shifting” to form clonal dominant tumors. We propose that the conservation mechanism, by which mtDNA sequences are maintained, appears to be achieved through mtDNA replication, which is remarkably faithful. This conclusion is based upon the observation that mtDNA sequence variation or lack thereof are present in phenotypically heterogenous cellular populations comprised of the progeny of a single cellular antecedent. Further studies such as direct replacement of mitochondria carrying a marked genome in a clonogenic cell and examination of the mtDNA from a sub- clonal population developed from such a cell is required for final proof.

## SUPPLEMENTARY MATERIALS



## Abbreviations

MtDNA: Mitochondrial Deoxynucleic Acid
SNP: Single Nucleotide Polymorphism
MMTV: Mouse Mammary Tumor-related Virus
G6PD: Glucose-6-phosphate Dehydrogenase
PGK: Phospho Glycerate Kinase
HUMARA: Human Androgen Receptor
LOH: Loss of Heterozygosity
DNA: Deoxynucleic Acid
RNA: Ribonucleic Acid
HVR2: Hypervariable Region 2
D Loop: Displacement Loop
NGS: Next Generation Sequencing
PCR: Polymerase Chain Reaction
ddPCR: Digital Droplet PCR
Trn-Pro: Transfer RNA- Proline
MtCo3: Mitochondrial Cyclooxygenase 3
Rrn1: Ribosomal RNA 1
DIN: DNA Integrity Number
NADH: Nicotinamide Adenine Dinucleotide
MtND1: Mitochondrial NADH Dehydrogenase 1
MtND5: Mitochondrial NADH Dehydrogenase 5
SIFT: Sorting Intolerable from Tolerable
MtCO1: Mitochondrial Cyclooxygenase 1
MtCytb: Mitochondrial Cytochrome b
HOG: Hyperplasia Outgrowths
ETC: Electron Transport Chain
ANOVA: Analysis of Variance

## References

[1] Linder D , Gartler SM . Glucose-6-Phosphate Dehydrogenase Mosaicism: Utilization as a Cell Marker in the Study of Leiomyomas. Science. 1965; 150:67–69. 10.1126/science.150.3692.67. 5833538

[2] Van Kamp H , Jansen R , Willemze R , Fibbe WE , Landegent JE . Studies on clonality by PCR analysis of the PGK-1 gene. Nucleic Acids Res. 1991; 19:2794. 10.1093/nar/19.10.2794. 2041762 PMC328217

[3] Mashal RD , Lester SC , Sklar J . Clonal analysis by study of X chromosome inactivation in formalin-fixed paraffin embedded tissue. Cancer Res. 1993; 53:46776–46779. 8402645

[4] Cui Z , Pan X , Wang Q . LOH detected by microsatellite markers reveals the clonal origin of recurrent laryngeal squamous cell carcinoma. PLoS One. 2014; 9:e111857. 10.1371/journal.pone.0111857. 25365429 PMC4218824

[5] Fliss MS , Usadel H , Caballero OL , Wu L , Buta MR , Eleff SM , Jen J , Sidransky D . Facile detection of mitochondrial DNA mutations in tumors and bodily fluids. Science. 2000; 287:2017–2019. 10.1126/science.287.5460.2017. 10720328

[6] Callahan R , Smith GH . The Mouse as a Model for Mammary Tumorigenesis: History and Current Aspects. J Mammary Gland Biol Neoplasia. 2008; 13:269. 10.1007/s10911-008-9094-4. 18712586

[7] Kordon EC , Smith GH . An entire functional mammary gland may comprise the progeny from a single cell. Development. 1998; 125:1921–1930. 9550724 10.1242/dev.125.10.1921

[8] Smith GH , Boulanger CA . Mammary stem cell repertoire: new insights in aging epithelial populations. Mech Ageing Dev. 2002; 123:1505–1519. 10.1016/S0047-6374(02)00114-8. 12425957

[9] Sanchez-Cespedes M , Parrella P , Nomoto S , Cohen D , Xiao Y , Esteller M , Jeronimo C , Jordan RC , Nicol T , Koch WM , Schoenberg M , Mazzarelli P , Fazio VM , et al. Identification of a Mononucleotide Repeat as a Major Target for Mitochondrial DNA Alterations in Human Tumors. Cancer Res. 2001; 61:7015–7019. 11585726

[10] Feeley KP , Bray AW , Westbrook DG , Johnson LW , Kesterson RA , Ballinger SW , Welch DR . Mitochondrial Genetics Regulate Breast Cancer Tumorigenicity and Metastatic Potential. Cancer Res. 2013; 75:4429–4436. 10.1158/0008-5472.CAN-15-0074. 26471915 PMC4610037

[11] Yadav N , Chandra D . Mitochondrial DNA Mutations and Breast Tumorigenesis. Biochim Biophys Acta. 2013; 1836:336–344. 10.1016/j.bbcan.2013.10.002. 24140413 PMC3891589

[12] Girolimetti G . Mitochondrial DNA Sequencing Demonstrates Clonality of Peritoneal Implants of Borderline Ovarian Tumors. Mol Cancer. 2017; 16:47. 10.1186/s12943-017-0614-y. 28241835 PMC5327524

[13] Chatterjee A , Mambo E , Sidransky D . Mitochondrial DNA mutations in human cancer. Oncogene. 2016; 25:4663–4674. 10.1038/sj.onc.1209604. 16892080

[14] Geurts-Giele WR , Gathier GH , Atmodimedjo PN , Dubbink HJ , Dinjens WN . Mitochondrial D310 Mutation as Clonal Marker for Solid Tumors. Virchows Arch. 2015; 467:595–602. 10.1007/s00428-015-1817-5. 26276353 PMC4656708

[15] Gaudio D , Fernandes DM , Schmidt R , Cheronet O , Mazzarelli D , Mattia M , O’Keefe T , Feeney RNM , Cattaneo C , Pinhasi R . Genome-Wide DNA from degraded Petrous Bones and the Assesment of Sex and Probable Geographic Origins of Forensics Cases. Sci Rep. 2019; 9:8226. 10.1038/s41598-019-44638-w. 31160682 PMC6547751

[16] Klinger CM , Karnkowska A , Herman EK , Hampl V , Dacks JB . Phylogeny and Evolution In: Walochnik J , and Duchêne M , eds. Molecular Parasitology. Vienna: Springer; 2016 10.1007/978-3-7091-1416-2_12.

[17] Arif IA , Khan HA , Bahkali AH , Al Homaidan AA , Al Farhan AH , Al Sadoon M , Shobrak M . DNA Marker Technology for Wildlife Conservation. Saudi J Biol Sci. 2011; 18:219–225. 10.1016/j.sjbs.2011.03.002. 23961128 PMC3730548

[18] Kirches E . MtDNA As a Cancer Marker: A Finally Closed Chapter? Curr Genomics. 2017; 3:255–267. 10.2174/1389202918666170105093635. 28659721 PMC5476953

[19] Callahan R , Mudunur U , Bargo S , Raafat A , McCurdy D , Boulanger C , Lowther W , Stephens R , Luke BT , Stewart C , Wu X , Munroe D , Smith GH . Genes affected by mouse mammary tumor virus (MMTV) proviral insertions in mouse mammary tumors are deregulated or mutated in primary human mammary tumors. Oncotarget. 2012; 3:1320–1334. 10.18632/oncotarget.682. 23131872 PMC3717796

[20] Coller HA , Khrapko K , Bodyak ND , Nekhaeva E , Herrero-Jimenez P , Thilly WG . High Frequency of Homoplasmic Mitochondrial DNA Mutations in Human Tumors can be Explained Without Selection. Nat Genet. 2001; 28:147–150. 10.1038/88859. 11381261

[21] Kim H , Komiyama T , Inomoto C , Kamiguchi H , Kajiwara H , Kobayashi H , Nakamura N , Terachi T . Mutations in the Mitochondrial ND1 Gene Are Associated with Postoperative Prognosis of Localized Renal Cell Carcinoma. Int J Mol Sci. 2016; 17:12–2049. 10.3390/ijms17122049. 27941608 PMC5187849

[22] Iommarini L , Kurelac I , Capristo M , Calvaruso MA , Giorgio V , Bergamini C , Ghelli A , Nanni P , Giovanni CD , Carelli V , Fato R , Lollini PL , Rugolo M , et al. M Different mtDNA mutations modify tumor progression in dependence of the degree of respiratory complex I impairment. Hum Mol Genet. 2014; 23:1453–1466. 10.1093/hmg/ddt533. 24163135

[23] Slaska B , Grzybowska-Szatkowska L , Nisztuk S , Surdyka M , Rozanska D . Mitochondrial DNA polymorphism in genes encoding ND1, COI and CYTB in canine malignant cancers. Mitochondrial DNA. 2015; 26:452–458. 10.3109/19401736.2013.840594. 24102599

[24] Lee HC , Chang CM , Chi CW . Somatic mutations of mitochondrial DNA in aging and cancer progression. Ageing Res Rev. 2010; 9:S47–S58. 10.1016/j.arr.2010.08.009. 20816876

[25] Carling PJ , Cree LM , Chinnery PF . The implications of mitochondrial DNA copy number regulation during embryogenesis. Mitochondrion. 2011; 11:686–692. 10.1016/j.mito.2011.05.004. 21635974

[26] Callahan R , Drohan W , Gallahan D , D’Hoostelaere L , Potter M . Novel class of mouse mammary tumor virus-related DNA sequences found in all species of Mus, including mice lacking the virus proviral genome. Proc Natl Acad Sci U S A. 1982; 79:4113–4117. 10.1073/pnas.79.13.4113. 6287466 PMC346587

[27] DeOme KB , Faulkin LJ Jr , Bern HA , Blair PB . Development of mammary tumors from hyperplastic alveolar nodules transplanted into gland-free mammary fat pads of female C3H mice. Cancer Res. 1959; 19:515–520. 13663040

[28] Daniel CW , DeOme KB , Young JT , Blair PB . The *in vivo* life span of normal and preneoplastic mouse mammary glands: a serial transplantation study. Proc Natl Acad Sci U S A. 1968; 61:53–60. 10.1073/pnas.61.1.53. 4301594 PMC285904

[29] Medina D . Serial transplantation of carcinogen-treated mammary nodule outgrowths. 3. Dissociation of carcinogen-induced cell variants by dose and chemical structure of carcinogen. J Natl Cancer Inst. 1973; 50:1555–1559. 10.1093/jnci/50.6.1555. 4717566

[30] Smith GH , Vonderhaar BK , Graham DE , Medina D . Expression of pregnancy-specific genes in preneoplastic mouse mammary tissues from virgin mice. Cancer Res. 1984; 44:3426–3437. 6430550

[31] Smith GH , Gallahan D , Zwiebek JA , Freeman SM , Bassin RH , Callahan R . Long-term *in vivo* expression of genes introduced by retrovirus- mediated transfer into mammary epithelial cells. J Virol. 1991; 65:6365–6370. 10.1128/jvi.65.11.6365-6370.1991. 1656102 PMC250361

[32] Bolger AM , Lohse M , Usadel B . Trimmomatic: A flexible trimmer for Illumina. Bioinformatics. 2014; 30:2114–20. 10.1093/bioinformatics/btu170. 24695404 PMC4103590

[33] McKenna A , Hanna M , Banks E , Sivachenko A , Cibulskis K , Kernytsky A , Garimella K , Altshuler D , Gabriel S , Daly M , DePristo MA . The Genome Analysis Toolkit: a MapReduce framework for analyzing next-generation DNA sequencing data. Genome Res. 2010; 20:1297–1303. 10.1101/gr.107524.110. 20644199 PMC2928508

[34] Van der Auwera GA , Carneiro MO , Hartl C , Poplin R , Del Angel G , Levy-Moonshine A , Jordan T , Shakir K , Roazen D , Thibault J , Banks E , Garimella KV , Altshuler D , et al. From FastQ data to high confidence variant calls: The Genome Analysis Toolkit best practices pipeline. Curr Protoc Bioinformatics. 2013; 43:11.10.1–33. 10.1002/0471250953.bi1110s43. 25431634 PMC4243306

[35] McLaren W , Gil L , Hunt SE , Riat HS , Ritchie GRS , Thormann A , Flicek P , Cunningham F . The Ensembl Variant Effect Predictor. Genome Biol. 2016; 17:122. 10.1186/s13059-016-0974-4. 27268795 PMC4893825

[36] Purcell SNB , Todd-Brown K , Thomas L , Ferreira MAR , Bender D , Maller J , Sklar P , de Bakker PIW , Daly MJ , Sham PC . PLINK: a tool set for whole-genome association and population-based linkage analysis. Am J Hum Genet. 2007; 81:559–75. 10.1086/519795. 17701901 PMC1950838

