# Supplementary material for: Sequence conservation of mitochondrial (mt)DNA during expansion of clonal mammary epithelial populations suggests a common mtDNA template in CzechII mice
Source: Oncotarget. 2020 Jan 14;11(2):161–74. doi: 10.18632/oncotarget.27429 (PMC6968779; doi:10.18632/oncotarget.27429)
Supplement: Supplementary file 1 [file oncotarget-11-161-s001.pdf]

Sequence conservation of mitochondrial (mt)DNA during expansion of clonal mammary epithelial populations suggests a common mtDNA template in CzechII mice

SUPPLEMENTARY MATERIALS

Supplementary Table 1: Summary of CzechII mitochondrial DNA analysis for mammary L12 normal and R12 normal, tumor and metastatic tumor tissue fragment outgrowths

Sample Name and Description	Mt Genome Coverage ≥ 50X	Mt Genome Median Coverage
**CzechII Non-Mammary (*n* = 1)**		
**Liver (Co.)**	99.90%	5070.0X
**CzechII Normal Mammary Outgrowths of R12 and L12 (*n* = 8)**		
**Lactating Gland (Co.)**	99.70%	756.0X
**L12 (Co.)**	99.60%	280.0X
**L12 1**	99.70%	385.0X
**L12 2**	99.60%	408.0X
**L12 3**	99.70%	1326.0X
**L12 4**	99.70%	348.0X
**R12 (Tgen. 2)**	82.50%	78.0X
**R12 (Tgen. 4)**	99.70%	399.0X
**CzechII Mammary Serially Transplanted (S.T) Tumor Outgrowths of R12 (*n* = 16)**		
**Tumor R12 Primary (Co.)**	99.70%	1320.0X
**Tumor R12 T1a (S.T.)**	99.80%	1426.0X
**Tumor R12 T1b (S.T.)**	99.80%	1908.0X
**Tumor R12 T2a (S.T.)**	99.80%	1367.0X
**Tumor R12 T2b (S.T.)**	99.90%	1388.0X
**Tumor R12 T3a (S.T.)**	100.00%	2279.0X
**Tumor R12 T3b (S.T.)**	0.00%	5804.0X
**Tumor R12 T4a (S.T.)**	99.80%	1958.0X
**Tumor R12 T5a (S.T.)**	99.40%	300.0X
**Tumor R12 T5b (S.T.)**	98.60%	304.0X
**Tumor R12 T6a (S.T.)**	99.70%	503.0X
**Tumor R12 T6b (S.T.)**	99.50%	302.0X
**Tumor R12 T7a (S.T.)**	100.00%	1481.0X
**Tumor R12 7b (S.T.)**	99.90%	2044.0X
**Tumor R12 T8 (S.T.)**	99.90%	16763.0X
**Unrelated Tumor 1 (S.T.)**	99.90%	5506.0X
**CzechII Mammary Tumor Serially Transplanted Metastatic Tumor Outgrowths of R12 (*n* = 5)**		
**Tumor R12 Lung Met. 1**	99.90%	8026.0X
**Tumor R12 Lung Met. 2**	99.90%	6761.0X
**Tumor R12 Lung Met. 3**	99.80%	3329.0X

**Tumor 12 Lung Met. 4**	99.90%	4568.0X
**Tumor R12 Lung Met. 5**	99.90%	14667.0X
**CzechII Mammary Serially Transplanted Hyperplasia, Tumor and Metastatic Tumor Outgrowths of CZN5 (*n* = 4)**		
**CZN5 Hyperplasia**	99.70%	1401.0X
**CZN5 Tumor**	99.90%	18310.0X
**CZN5 Independent Tumor 1**	99.90%	12459.0X
**CZN5 Independent Tumor 2**	99.90%	7691.0X

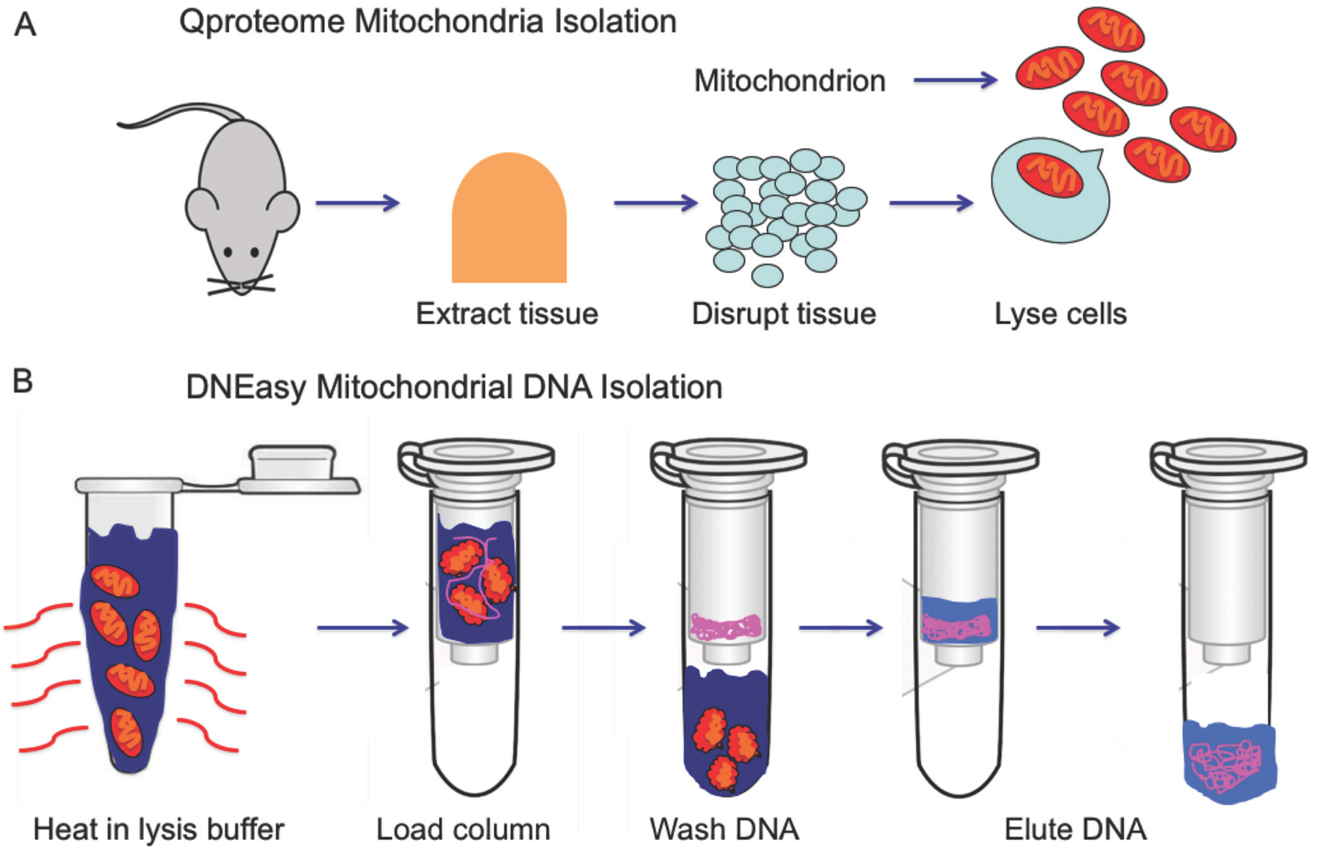

Supplementary Figure 1: Mitochondrion and mitochondrial DNA isolation methodology. Cartoon schematic depicts novel mitochondrial isolation assay designed and optimized for fresh and snap frozen mammary tumor CzechII tissue fragments (**A**). Qiagen's' DNeasy isolation kit was used to isolated intact CzechII mitochondrial DNA from isolated mitochondria, as depicted in cartoon (**B**).
